# Assessment of mechanical weeders in paddy fields: A study on operational effectiveness in Bangladesh

**DOI:** 10.1016/j.heliyon.2025.e42639

**Published:** 2025-02-12

**Authors:** Subrata Paul, Bidhan Chandra Nath, Md. Durrul Huda, Md. Golam Kibria Bhuiyan, Haimonti Paul

**Affiliations:** Farm Machinery and Post-Harvest Technology (FMPHT) Division, Bangladesh Rice Research Institute (BRRI), Gazipur, 1701, Bangladesh

**Keywords:** Mechanical weeders, Paddy fields, Weeding efficiency, Power weeder, Rice, Weed management

## Abstract

Rice is a vital crop for food security in Bangladesh, occupying 75 % of its agricultural land. Weed management is crucial for maintaining rice yields because weeds compete for nutrients, water, and space, leading to yield losses ranging from 15 % to 60 %. Traditional hand weeding is labor-intensive and costly, driving the need for efficient, sustainable alternatives. This study assessed the operational effectiveness of five weeding technologies: BRRI Power Weeder (PW), BRRI Weeder (BW), BRRI Conical Weeder (CW), BRRI Double Row Weeder (DW), and Manual Hand Weeding (HW). Field trials were conducted at the Bangladesh Rice Research Institute (BRRI) and farmers' fields in Jogitola, Gazipur, during the 2022 Boro season. A comprehensive assessment of five distinct weeding technologies for rice cultivation in Bangladesh, considering their operational performance under research and field conditions. Essential performance indicators were evaluated-field capacity, weeding efficiency, tiller damage, and fuel consumption. The results revealed that the BRRI Power Weeder had the highest field capacity (669 m^2^/h) but caused more crop damage and had lower field efficiency due to turning delays. In contrast, manually operated weeders like BW and CW showed moderate field capacities (247 m^2^/h and 228 m^2^/h, respectively) with better crop safety and higher field efficiency. Manual hand weeding, while labor-intensive with the lowest field capacity (100 m^2^/h), caused minimal tiller damage and was most effective for small-scale fields. The findings indicate that mechanical weeders offer labor savings and improved efficiency, but crop safety and field conditions must guide technology selection. The commitment to balancing operational efficiency with crop safety is crucial for sustainable rice production in Bangladesh.

## 1 Introduction

Rice (*Oryza sativa* L.) is a fundamental staple food crop, sustaining more than half of the global population [1]. It is especially significant in Asia, where it constitutes a vital part of the diet and economy. Bangladesh is one of the world's leading rice-producing countries, with rice accounting for about 75 % of the country's agricultural land and contributing significantly to the national food supply [2]. Sustainable rice production is important to the region's food security and economic stability. However, various factors often threaten rice yields, including weed infestation, which competes with rice plants for essential resources such as nutrients, water, light, and space [3]. This reduces yield significantly if not effectively managed.

Weeds are a significant constraint in rice production, capable of causing yield losses between 15 % and 60 % depending on the severity of infestation and management practices [4]. Weeds are the most significant challenge in crop cultivation, leading to substantial losses in crop yield [5]. Traditional weed control methods in rice fields, mainly manual hand weeding, remain prevalent in many developing countries, including Bangladesh. Hand weeding is labor-intensive, time-consuming, and often leads to high labor costs, accounting for up to 30–40 % of the total production costs [6]. Furthermore, the availability of agricultural labor is declining due to urbanization and migration, making manual weeding increasingly impractical and unsustainable [7]. As a result, there is an urgent need for alternative weed management strategies that are efficient, cost-effective, and sustainable, as weed control significantly impacts crop production costs, making effective management essential for the economic sustainability of rice farming in Bangladesh [8].

While precise and effective in minimizing crop damage, traditional hand weeding is physically demanding, time-consuming, and increasingly costly due to rising labor wages. Mechanical weeders present a promising alternative, offering significant labor savings and improved efficiency by covering larger areas in less time [9]. These machines are specifically designed to operate between rice rows, uprooting or burying weeds with minimal crop damage while providing benefits such as better soil aeration and reduced compaction, which enhance root growth and overall plant health [10]. However, their effectiveness can be influenced by factors such as soil type, weed density, and field conditions, and they may cause more crop damage compared to manual methods [11]. The decision to adopt hand or mechanical weeding depends on farm size, labor availability, and specific farming needs. For smallholder farmers, affordable and user-friendly mechanical weeders could reduce production costs, increase productivity, and improve profit margins [12]. Promoting such technologies supports sustainable farming practices and aligns national priorities to enhance agricultural efficiency and food security [13]. Addressing these economic and operational challenges, mechanical weeders can play a vital role in modernizing rice farming in Bangladesh by reducing dependency on manual labor while ensuring sustainable farming practices [14].

Adopting mechanical weeders offers a transformative shift beyond immediate labor savings in smallholder farming systems [8]. Farmers can reallocate their time and energy towards crucial activities like crop diversification, livestock rearing, and infrastructure improvements by reducing reliance on manual labor. This leads to increased income diversification and enhanced livelihoods. The timely and efficient weeding enabled by mechanical weeders boosts crop growth and yield and enhances food security. Moreover, by optimizing resource utilization and minimizing labor inputs, these machines contribute to more sustainable and resilient agricultural practices, particularly in resource-constrained environments. As labor shortages and rising wages become pressing challenges, mechanical weeders offer a compelling solution, increasing efficiency, precision, and timeliness in weeding operations. Beyond labor savings, these machines minimize physical strain on farmers and improve soil health through better aeration and reduced compaction, fostering more sustainable farming practices. By addressing these critical needs, adopting mechanical weeders can revolutionize smallholder farming, enhancing productivity, economic resilience, and efficient resource utilization.

Weed management in Bangladeshi rice cultivation demands a multifaceted approach. While organic methods like mulching and crop rotation promote sustainability, they can be less effective against heavy infestations [15]. Inorganic methods, such as herbicides, offer rapid control but pose environmental and human health risks [16,17]. Mechanical weeders, while potentially damaging crops, significantly increase efficiency and reduce labor. Combining mechanical weeding and carefully selected organic practices, an integrated approach offers the most promising solution [18]. This approach optimizes weed control by balancing efficiency, environmental sustainability, and economic viability for smallholder farmers, ensuring the long-term sustainability of rice production systems. Recent research has demonstrated the potential benefits of using mechanical weeders in rice cultivation. For example, a study by Hussain et al. [19] found that mechanical weeders could achieve weeding efficiencies of up to 85 %, comparable to or better than manual weeding, while significantly reducing labor input. Similarly, Ahmed et al. [6] reported that mechanical weeders in Bangladesh reduced weeding time by 50–70 % compared to manual methods, leading to substantial savings in labor costs. Adopting mechanical weeders has also increased crop yields due to more effective weed control and less competition [20].

The Bangladesh Rice Research Institute (BRRI) has developed and promoted mechanical weeders tailored to the unique conditions of Bangladeshi rice fields. These include the BRRI power weeder (PW), BRRI weeder (BW), BRRI conical weeder (CW), and Double-row weeder (DW), each designed to accommodate local field conditions, crop spacing, and resource availability, making them suitable for smallholder farmers [21]. The BRRI power weeder, adapted from a Korean design, operates with a small petrol engine, covering 60 cm in a single pass, significantly reducing labor and time requirements (www.brri.gov.bd). However, the performance of mechanical weeders depends on factors such as design quality, field conditions, weed density, and operator skill. Power-operated weeders offer higher field capacities and faster operation but may risk crop damage if misused [22]. In contrast, manually operated weeders, such as the BRRI weeder and conical weeder, provide precision and control, making them ideal for smaller fields or densely planted areas [23]. Soil conditions, water levels, and weed types also affect performance; for instance, conical weeders are effective in soft, water-saturated soils, while double-row weeders excel in densely planted fields by efficiently controlling weeds without disturbing the rice plants [9].

In Bangladesh, most weeding-related studies focus solely on the development and performance of individual weeders [8,21,24,25], with little attention to comparative evaluations of diverse weeding practices, such as hand, semi-manual, and mechanical methods. This study addresses this gap by thoroughly evaluating five weeding technologies for rice cultivation in Bangladesh and analyzing their operational performance in controlled research settings and real-world field conditions. Unlike earlier studies, it assesses critical performance indicators, including field capacity, weeding efficiency, tiller damage, and fuel consumption, tackling the dual challenges of labor-intensive hand weeding and crop safety. By comparing mechanical and manual methods, the study highlights trade-offs between efficiency and crop protection, offering actionable insights for farmers to select the most suitable technologies for specific field conditions. Furthermore, by identifying the most efficient and effective practices, this research aims to support adopting best practices in weed management, enhancing productivity, reducing labor costs, and promoting sustainable agriculture in rice cultivation. The findings are expected to inform policymakers, extension services, and farmers, contributing to improved rice production and strengthened food security in Bangladesh.

## 2 Materials and methods

**Fig. 1:**
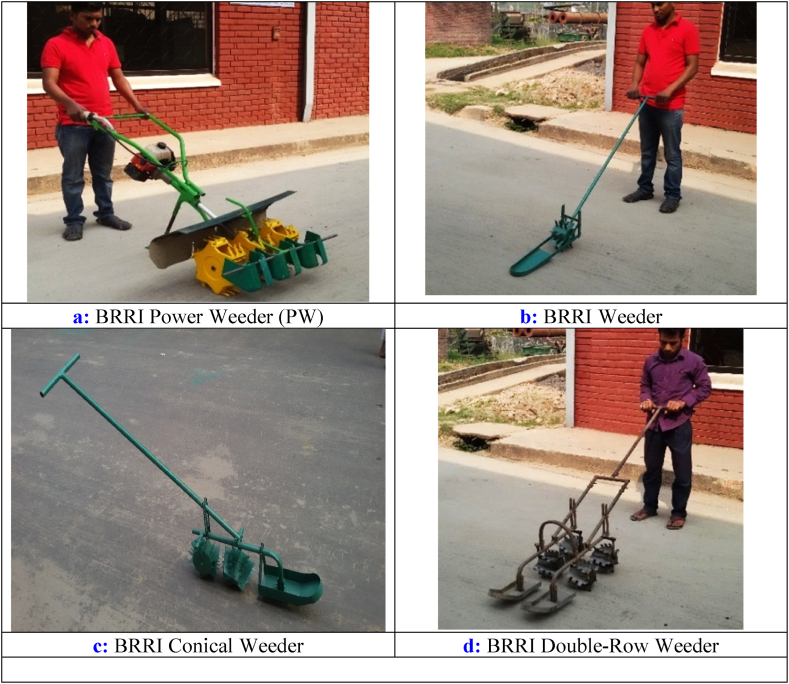
Various types of weeders are used for rice weeding in Bangladesh.

This study was conducted in Bangladesh's Bangladesh Rice Research Institute (BRRI) experimental paddy field. Different weeders were evaluated during the Boro/2022 season at the BRRI experimental field and farmer's field at Jogitola, Gazipur, where the soil texture was mainly similar. Both field settings considered BRRI dhan28, a mega variety of Bangladesh. This study utilized four widely available and popular weeding technologies: the BRRI-developed power weeder (PW), BRRI weeder (BW), BRRI conical weeder (CW), and Double-row weeder (DW). Manual hand weeding (HW) and four technologies were also included as the control treatment. Fig. 1 (a, b, c, and d) presents pictorial views of the mechanical weeders used in the experiment. A power tiller prepared the paddy field using conventional tillage practice under flooding conditions. The hand transplanting method was applied to transplantation in the research field. Transplanting was done in rows at 20 cm fixed intervals. Because of the short hill spacing, laborers removed the weeds between them, and weeding machines controlled the weeds between the rows. RCB design was applied with three replications. All treatments were standardized to ensure accuracy, with manual transplantation conducted at a hill-to-hill spacing of 20 × 20 cm, using 2–3 seedlings per hill while maintaining uniform variety, fertilizer application, and crop management across all treatments. The first weeding was conducted at 20 and 25 days after transplanting (DAT), and the second weeding date was 34 and 40 DAT in the BRRI research field and farmer's field in Jogitola, Gazipur, respectively.

### 2.1 Description of the different weeding technologies

#### 2.1.1 BRRI-developed power weeder

Adapting the Korean power weeder for use in Bangladesh is a significant development in agricultural technology. Tailoring the machine to local conditions and constructing it in a regional workshop supports the economy and ensures that the technology is accessible and suitable for the farmers. Using a small petrol engine makes it a practical choice for the small-scale farms typically in the region (Fig. 1). Its ability to cover 60 cm width in a single pass increases efficiency, reducing the time and labor required for weeding. The design consideration for weeding spikes and rotors demonstrates an understanding of the need for durable, lightweight equipment in agricultural operations. With a total weight of 22 kg, the machine's portability is an added advantage, making it user-friendly for a diverse workforce. This innovation exemplifies how localized engineering can lead to practical solutions in agriculture, enhancing productivity while considering farm machinery's ergonomic and economic aspects.

#### 2.1.2 BRRI weeder

The BRRI weeder is a manual push-pull agricultural tool designed to manage weeds in paddy fields efficiently. Its optimal use is for rows spaced 20 cm apart, allowing it to cover an equivalent field width with each pass. The compact design, featuring a 15 cm wide rotor with 12 single spikes, makes it manageable for a single individual to operate (Fig. 1b). Weighing only 3.5 kg, it is a lightweight solution that maintains crop health and productivity by efficiently controlling unwanted plant growth.

#### 2.1.3 BRRI conical weeder

The BRRI conical weeder represents a significant advancement in agricultural tools, specifically designed to enhance the efficiency of weed management in transplanted fields. Its innovative design, featuring dual conical rotors with alternating smooth and serrated blades, allows for effective uprooting and burying weeds with minimal effort. The lightweight nature of the weeder, at just 5.4 kg, ensures ease of use over extended periods, while its coverage width of 13–15 cm is optimal for single-row weeding (Fig. 1c). Developed within the FMPHT divisional research workshop, this tool exemplifies the integration of practical design and agricultural science to meet the needs of modern farming practices.

#### 2.1.4 BRRI double row weeder

The manually operated double-row weeder is a significant innovation in agricultural tools designed to manage weeds in rice fields efficiently. Its push-pull operation and the ability to work the top 3 cm of soil make it an effective solution for uprooting weeds without disturbing the deeper soil layers, which can be beneficial for preserving soil structure and moisture. The compact design, with a rotor length of 12.5 cm and blades measuring 4.5 cm by 2 cm, ensures precision in targeting weeds between the rows (Fig. 1d). The main frame's capacity holds four rotary-type rotors, each equipped with six serrated blades, allowing for a thorough and consistent weeding process. The helical arrangement of the blades on the drum is particularly innovative, as it ensures that weeds are uprooted and buried, which can hinder their regrowth. With an average weight of 7.5 kg and an effective operational width of 35–37 cm, this weeder is manageable and efficient, covering a substantial area without causing fatigue to the operator. This tool exemplifies practical engineering that can lead to more sustainable and labor-efficient farming practices.

### 2.2 Evaluation procedure

#### 2.2.1 Site characterization and experimental setup

A field trial was carried out at the Bangladesh Rice Research Institute (BRRI) and in Jogitola, Gazipur, to assess the efficacy of weeding in a rice field. The field preparation involved a single power tiller operation. Hand transplanting was employed for planting, with a row spacing maintained at 20 cm. The predominant weed type in the experimental field was grassy weeds. The field conditions included standing water levels of three to 6 cm. The rice plants exhibited a height variation between 22 and 32 cm, as detailed in Table 1. This standard description provides a clear overview of the field trial conditions.

**Table 1:** Condition of the field during the first weeding.

Parameters/Items	Location	
	BRRI Research field	Jogitola, Gazipur
Depth of standing water (cm)	3–5	4–6
Type of predominant weed	*Scirpus maritimus*	*Scirpus maritimus*
Size of weeds (cm)	15–18	17–21
Stage of maturity of crop, days	20	25
Row spacing of crop, cm	20	20
Plant height (cm)	22–25	28–32

#### 2.2.2 Data collation

Walking speed was measured to determine the theoretical field capacity of the weeder. The total time of field operation was recorded to calculate the actual field capacity, accounting for turning loss, operator's loss, and machine adjustment and troubleshooting loss during field operation. The number of weeds and tillers were counted in pre-selected 1 m^2^ areas before and after the operation. Weed biomass was measured by collecting and drying weeds from 1 m^2^ areas at 75 °C for 48 h. We used a specific formula to calculate weeding capacity, weeding efficiency, and the number of damaged tillers/hills.

### 2.3 Machine parameters

#### 2.3.1 Travel/walking speed (km/h)

During the weeding operation, the machine's travel speed was quantified by recording the duration required to travel 10 m. Each method was repeated five times to ensure accuracy, and the mean value was computed from the collected data. The timings were meticulously noted in seconds with a digital stopwatch to maintain precision.

#### 2.3.2 Effective working width (mm)

The effective width of the weeding process and the weeder itself are identical. The weeder is tested to have an effective width slightly narrower than its theoretical width. To ascertain the exact coverage area of the weeding, a 5-m steel tape measure was employed. This precise measurement ensures that the weeder's performance can be accurately assessed and compared to its intended design specifications.

#### 2.3.3 Actual field capacity

In the research areas, measurements were made of the developed weeder's actual field capacity. To determine the weeder's actual field capacity, the machine running duration considered the weeder's turning time, operator time, adjustment time, re-starting time, and other parameters. According to Hunt [26], it is the machine's average field coverage rate ratio to its total running time (Equation (1)). Therefore, [26]

(1) $$C=\frac{A}{T}$$

where,

C= Actual field capacity in ha/h.

A = Area of weeding in hector.

T = Time of weeding in h.

#### 2.3.4 Theoretical field capacity (ha/h)

The rate of field coverage attained if the weeder runs continuously is known as theoretical field capacity. In theory, it is predicated on speed and width. The relationship shown below was used to compute the theoretical field capacity (Equation (2)) [27].

(2) $$\text{Theoretical}\;\text{field}\;\text{capacity}=\frac{Width\;of\;the\;implement\;\left(m\right)\;x\;speed\;of\;operation\;\left(km/h\right)}{10}$$

#### 2.3.5 Field efficiency (%)

The field efficiency was calculated using the equation (Equation (3))[28]:

(3) $$\text{Field}\;\text{efficiency}\;\left(\%\right)=\frac{Actual\;field\;capacity\;\left(\frac{ha}{h}\right)}{Theoritical\;field\;capacity\;\left(\frac{ha}{h}\right)}\;x\;100$$

#### 2.3.6 Weeding efficiency

Finding the average number of weeds per square meter area before beginning any weeding is important. Five days after the end of the weeding test, counting the number of weeds left outside per square meter is also possible. The following calculations can be used to calculate the weeder's efficiency, and the difference between the two will show how many weeds were eradicated (Equation (4)) [29].

(4) $$W\text{eeding}\;\text{efficiency}\;\left(\text{WE}\right)=\frac{Number\;ofweedes\;eliminated\;per\;m^{2}}{Total\;number\;of\;weeds\;present\;per\;m^{2}}x\;100=\frac{W1‐W2}{W1}\;X\;100$$

where,

WE = Efficiency of weeding.

*W*_*1*_= Population of weeds before the operation.

*W*_*2*_= Population of weeds after the operation.

#### 2.3.7 Damaged tiller rate

The percentage of rice tiller breakage was determined using the following equation (Equation (5))[30]:

(5) $$DTR=\frac{T1‐T2}{T1}\;X\;100$$

where,

DTR = Damage of tiller in percentage.

*T*_*1*_ = Tiller number before weeding.

*T*_*2*_ = Tiller number after weeding.

### 2.4 Data analysis

This study uses IBM SPSS statistical package software version 27 (IBM corporate, Armonk, New York) to analyze experimental data. Treatment means were considered significantly different if P-value, P < 0.05.

## 3 Results and discussion

### 3.1 Field operation and performance

**Fig. 2:**
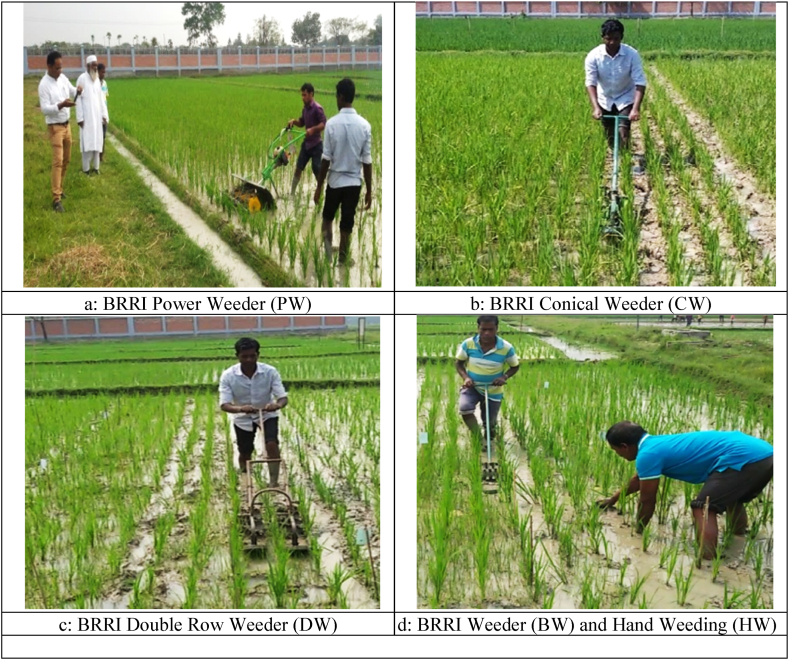
Weeding activities during the first weeding.

**First weeding*:*** The first weeding at the BRRI research field and Jogitola, Gazipur, was a critical step in the cultivation process. The standing water depth was carefully maintained at both locations between 3-5 cm and 4–6 cm to facilitate the weeding technologies. At 20 and 25 days of maturity, the crops were at a crucial growth stage, with plant heights ranging from 22 to 25 cm and 28–32 cm, respectively. This early intervention was essential for optimal growth and yield. The study examined five weeding technologies: the BRRI-developed power weeder (PW), BRRI weeder (BW), BRRI conical weeder (CW), BRRI Double row weeder (DW), and Manual hand weeding (HW). The results from this first weeding with different technologies are shown in Table 2, Table 3. The operational activities are also shown in Fig. 2.

**Table 2:** Weeding performance of first weeding with different weeding technologies.

Parameter	Location									
	BRRI research field					Farmers field, Jogitola, Gazipur				
	HW	BW	PW	CW	DW	HW	BW	PW	CW	DW
Weeding efficiency (%)	88.73^a^	70.54^b^	80.38^a^	77.37^a^	74.4^c^	91.02^a^	75.15^b^	81.43^a^	82.14^a^	79.35^c^
Weeds remain (%)	11.26	29.46	19.62	22.63	25.6	8.98	24.85	18.57	17.86	20.65
Tiller damage (%)	1.18	1.29	2.75	1.69	2.35	1.17	1.66	2.81	1.92	2.70
Revive of weeds after four days (%)	17.99	26.61	32.26	20.22	23.62	19.39	26.94	34.90	21.62	24.35
Revive of weeds after ten days (%)	60.85	82.5	78.76	62.33	76.68	63.59	84.16	81.85	67.74	76.68
Weeds biomass during weeding, gm/m^2^ (%)	27.37	31.22	35.43	29.38	25.52	32.46	34.23	30.88	28.34	27.45

Note.

The average value of three replications (N = 3) is presented in the Table.

Superscript letters of alphabets indicate that means followed by the same letter do not significantly differ at p = 0.05.

HW = Hand weeding; BW = BRRI weeder; PW = BRRI Power weeder; CW = BRRI Conical weeder and DW= BRRI Double row weeder.

**Table 3:** Field efficiency of first weeding with different weeding technology.

Parameter	Location									
	BRRI research field					Farmers field, Jogitola, Gazipur				
	HW	BW	PW	CW	DW	HW	BW	PW	CW	DW
Total operating time, min	18.12	7.09	2.07	7.41	4.55	18.0	7.34	2.79	7.45	4.47
Actual operating time, min	18.12	5.77	1.85	6.0	3.7	18.0	5.93	1.88	6.02	3.61
Walking speed, m/h	–	1480	1523	1423	1131	–	1401	1469	1381	1134
Theoretical capacity, m^2^/h	98	296	914	285	452	100	280	881	276	454
Actual field capacity, m^2^/h	98	239	614	229	368	100	226	593	221	366
Field Efficiency, %	–	81.14	67.3	80.72	81.39	–	80.74	67.52	80.48	80.65

Note.

The average value of the three replications is presented in the Table.

HW = Hand weeding; BW = BRRI weeder; PW = BRRI Power weeder; CW = BRRI Conical weeder and DW= BRRI Double row weeder.

**Fig. 3:**
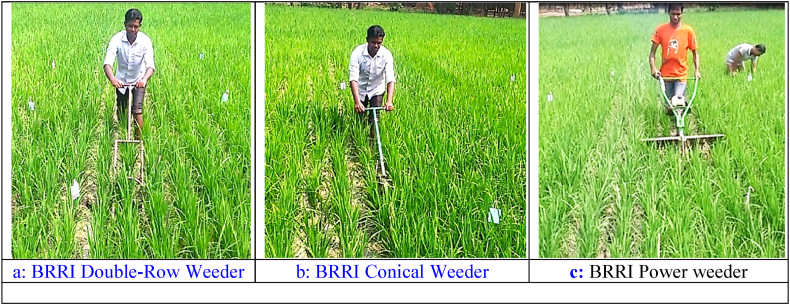
Weeding activities during the second weeding.

**Second weeding:** The data in Table 4, Table 5 offer a comprehensive overview of the outcomes from the second weeding, executed using various weeding technological approaches. These tables detail each method's effectiveness, efficiency, and economic implications. Meanwhile, Fig. 3 visually represents operational activities, including timelines, resource allocation, or procedural steps, offering a clear and structured understanding of the field operations conducted.

**Table 4:** Weeding performance of second weeding with different weeding technology.

Parameter	Location									
	BRRI research field					Farmers field, Jogitola, Gazipur				
	HW	BW	PW	CW	DW	HW	BW	PW	CW	DW
Weeding efficiency (%)	88.5^a^	73.2^b^	80.0^a^	83.8^a^	80.99^a^	93.1^a^	72.8^b^	81.5^a^	82.2^a^	79.6^a^
Weeds remain (%)	11.43	26.72	20.0	16.13	19.01	6.96	27.2	18.52	17.83	20.34
Tiller damage (%)	2.53	4.76	7.5	4.82	5.12	3.65	4.49	8.60	4.71	5.62

Note.

The average value of three replications (N = 3) is presented in the Table.

Superscript letters of alphabets indicate that means followed by the same letter do not significantly differ at p = 0.05.

HW = Hand weeding; BW = BRRI weeder; PW = BRRI Power weeder; CW = BRRI Conical weeder and DW= BRRI Double row weeder.

**Table 5:** Field efficiency of second weeding with different weeding technology.

Parameter	Location									
	BRRI research field					Farmers field, Jogitola, Gazipur				
	HW	BW	PW	CW	DW	HW	BW	PW	CW	DW
Total operating time, min	16	6.1	2.13	7.0	4.1	16.5	6.5	2.2	7.3	4.5
Actual operating time, min	16	4.7	1.4	5.4	3.2	16.5	5.0	1.5	5.7	3.38
Walking speed, m/h	–	1777	1988	1547	1305	–	1626	1808	1427	1204
Theoretical capacity, m^2^/h	102	355	1193	309	522	98	325	1162	285	481
Actual field capacity, m^2^/h	102	273	730	238	397	98	250	740	222	361
Field Efficiency, %	–	76.90	61.19	77.02	76.05	–	76.92	63.68	77.77	75.05

Note.

The average value of three replications (N = 3) is presented in the Table.

HW = Hand weeding; BW = BRRI weeder; PW = BRRI Power weeder; CW = BRRI Conical weeder and DW= BRRI Double row weeder.

### 3.2 Weeding efficiency

**Fig. 4:**
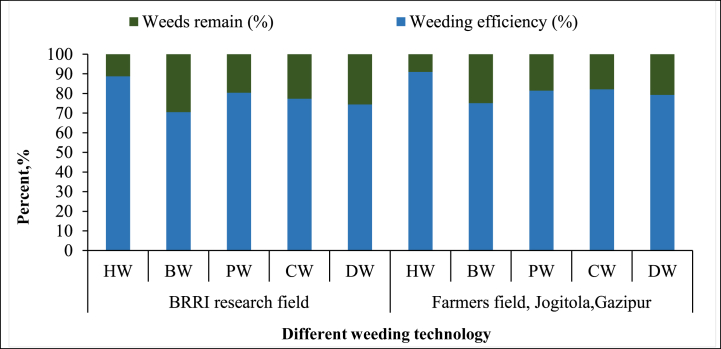
Weeding efficiency of different weeding during first weeding technology. Note: HW = Hand weeding; BW = BRRI weeder; PW = BRRI Power weeder; CW = BRRI Conical weeder and DW= BRRI Double row weeder.

**First weeding:** The first weeding Fig. 4 results show varying efficiencies across technologies. “Hand Weeding (HW)” had the highest efficiency in both the BRRI research field (88.73 %) and the farmer's field (91.02 %), confirming its precision and effectiveness. The “BRRI Power Weeder (PW)” performed well, with efficiencies of 80.38 % and 81.43 %, but struggled with taller weeds in the farmer's field, consistent with Ranji et al. [31]. The “BRRI Conical Weeder (CW),” however, outperformed the PW in the farmer's field (82.14 %), likely due to its adaptability to varied weed growth [32]. The “BRRI Double Row Weeder (DW)” showed moderate efficiency (74.4 % in the research field and 79.35 % in the farmer's field), while the “BRRI Weeder (BW)” had the lowest efficiency, with 70.54 % in the research field and 75.15 % in the farmer's field. This aligns with Cherati et al. [33], who found manual weeders less effective in dense weed conditions. These results underscore the importance of selecting the appropriate weeder based on weed density and field conditions.

**Fig. 5:**
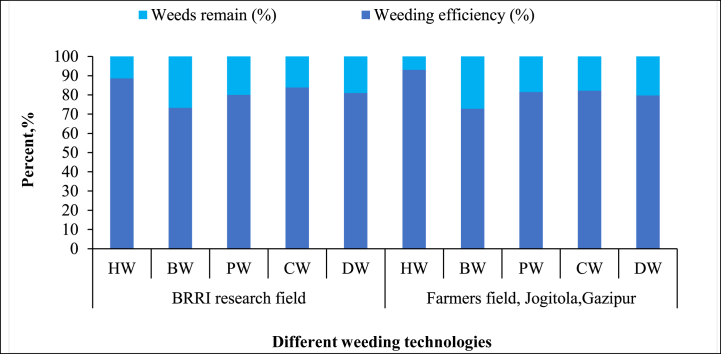
Weeding efficiency of different weeding technologies during second weeding. *Note:* HW = Hand weeding; BW = BRRI weeder; PW = BRRI Power weeder; CW = BRRI Conical weeder and DW= BRRI Double row weeder.

**Second weeding:** Weed density, equipment or machine performance, operator proficiency, and weed maturity influenced the second weeding operation. “Hand Weeding (HW)” maintained the highest weeding efficiency in both the BRRI research field (88.57 %) and the farmer's field in Jogitola (93.04 %) (Fig. 5). This aligns with Fennimore et al. [34], who emphasized the precision of manual weeding, particularly in the later stages of weed maturity. Manual methods are effective in densely weeding fields, allowing operators to selectively remove weeds without damaging crops. The “BRRI Conical Weeder (CW)” also showed high efficiency in both fields (83.87 % and 82.17 %, respectively). The “Power Weeder (PW)” demonstrated reasonable efficiency, particularly in the farmer's field (81.48 %), but slightly lower performance in the BRRI field (80.00 %). Its lower efficiency compared to the Conical Weeder is likely due to difficulties in uprooting mature weeds, as reported by Devanathan et al. [35], who observed that power weeders struggle with more established weed growth. Lastly, the “BRRI Double Row Weeder (DW)” exhibited moderate efficiency (80.99 % in the BRRI field and 79.66 % in the farmer's field). However, its slightly lower performance, particularly with mature weeds, indicates it may not be as effective in managing well-established weed populations, consistent with Islam et al. [36].

### 3.3 Tiller damage

**Fig. 6:**
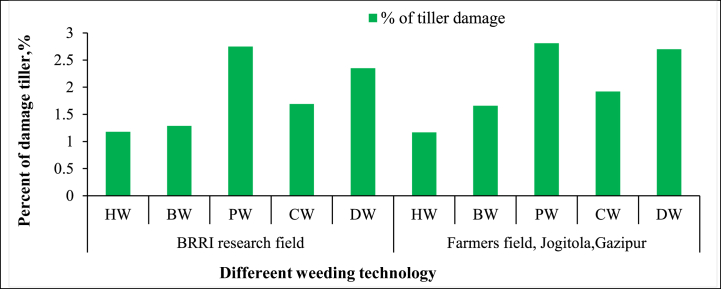
Percent of tiller damage from different weeding technologies. *Note:* HW = Hand weeding; BW = BRRI weeder; PW = BRRI Power weeder; CW = BRRI Conical weeder and DW= BRRI Double row weeder.

**First weeding:** In the first weeding, tiller damage varied significantly among the different technologies. “Hand Weeding (HW)” resulted in the most minor crop disturbance, with 1.18 % damage in the BRRI research field and 1.17 % in the farmer's field (Fig. 6). This aligns with the findings of Paul et al. [5], who emphasized the precision of manual weeding in minimizing crop damage, making it an optimal choice for smallholder farmers focused on crop preservation. The “BRRI Power Weeder (PW)” caused the most tiller damage, with 2.75 % in the BRRI research field and 2.81 % in the farmer's field. Islam et al. [37] also observed higher crop damage in mechanized weeders due to their aggressive nature and less control, especially in denser conditions. While efficient, the power weeder's mechanical action leads to a higher likelihood of crop disruption. The “BRRI Conical Weeder (CW)” recorded moderate damage rates, with 1.69 % in the research field and 1.92 % in the farmer's field. This reflects its efficiency in uprooting weeds while causing some crop disturbance, as noted by Ahiduzzaman and Islam [38]. The “BRRI Double Row Weeder (DW)” caused more damage than manual methods, with 2.35 % in the research field and 2.70 % in the farmer's field. The higher tiller damage reflects its aggressive weeding action, especially in denser weed patches like Manawaduge et al. [39]. The “BRRI Weeder (BW)” caused relatively low tiller damage, with 1.29 % in the research field and 1.66 % in the farmer's field. This confirms its status as a lower-impact mechanical option but still less gentle than hand weeding.

**Fig. 7:**
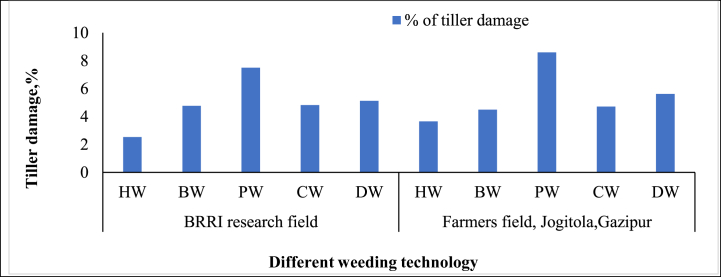
Percent of tiller damage from different weeding technologies. *Note:* HW = Hand weeding; BW = BRRI weeder; PW = BRRI Power weeder; CW = BRRI Conical weeder and DW= BRRI Double row weeder.

**Second weeding:** In the operation, tiller damage varied significantly among the different weeding technologies. The “BRRI Power Weeder (PW)” exhibited the highest level of tiller damage, with rates of 7.5 % in the BRRI research field and 8.60 % in the farmers' field (Fig. 7). This aligns with the findings of Islam et al. [40], who noted that the aggressive action of mechanized weeders like the PW can disturb the weeds and rice tillers, particularly in fields with dense or mature crops. The higher tiller damage is attributed to the weeder's mechanical components, which can damage crops during operation. In contrast, “Hand Weeding (HW)” caused the most minor tiller damage, with rates of 2.53 % in the BRRI research field and 3.65 % in the farmers' field. These results are consistent with Nath et al. [21], who reported that manual weeding offers better precision and control, minimizing crop damage even in dense planting conditions. The precision of hand weeding makes it an optimal choice for farmers who prioritize crop safety over operational speed. The “BRRI Weeder (BW),” “BRRI Conical Weeder (CW),” and “BRRI Double Row Weeder (DW)” caused moderate tiller damage, ranging from 4.49 % to 5.62 % in both field types. These findings reflect the trade-off between efficiency and crop safety, as observed by Shakya et al. [32], who noted that while these weeders provide faster weeding, they can still cause notable tiller damage due to their mechanical operation. The “Conical Weeder,” in particular, demonstrated a relatively balanced performance with moderate damage, aligning with Dilipkumar et al. [4], who found it to be an effective tool that balances weed removal efficiency with reasonable crop protection.

### 3.4 Weeds revive

**Fig. 8:**
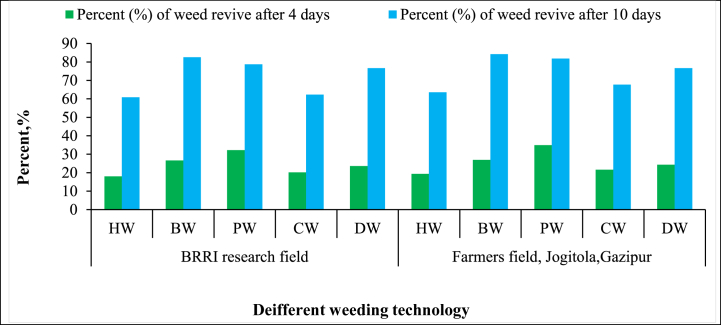
Weed revive after four days and ten days of different weeding technologies. *Note:* HW = Hand weeding; BW = BRRI weeder; PW = BRRI Power weeder; CW = BRRI Conical weeder and DW= BRRI Double row weeder.

The analysis of weed recovery four days post-weeding highlighted the varying effectiveness of different weeding technologies. The “BRRI Power Weeder” exhibited the highest weed revival rates, with 32.26 % in the BRRI research field and 34.90 % in the farmer's field (Fig. 8). This higher percentage of weed recovery can be attributed to the power weeder's inability to completely uproot taller, more mature weeds, which allows them to regrow. Bhuiyan et al. [41] state that power-operated weeders often struggle with deep-rooted and older weeds, especially in fields where weeds have advanced. On the other hand, “Hand Weeding (HW)” demonstrated the lowest weed revival rate, indicating its superior effectiveness in completely removing weeds. The precision of hand weeding ensures that weeds are uprooted entirely, reducing the likelihood of regrowth. As Monaco et al. [42] highlighted, manual weeding remains one of the most effective methods for controlling weed recovery due to its ability to target weeds at the root level. The “BRRI Conical Weeder (CW)” showed a significantly lower percentage of weed revival compared to other mechanical methods, with the dual-rotor design playing a pivotal role in effectively uprooting and burying weeds. Kumar et al. [43] noted that the conical weeder's design allows for deeper soil penetration, ensuring more complete removal of weeds and reducing the chances of regrowth. After ten days of post-weeding, the BRRI conical weeder outperformed other technologies, proving the most effective mechanical method in both fields. In contrast, the “BRRI Weeder (BW)” demonstrated higher weed revival after ten days, indicating that its effectiveness decreased over time. Although it initially provides moderate control, its ability to prevent long-term weed recovery is lower than the Conical Weeder and Hand Weeding, as corroborated by Peruzzi et al. [44], who found that manually operated weeders are less effective in controlling mature populations.

### 3.5 Field capacity

**Fig. 9:**
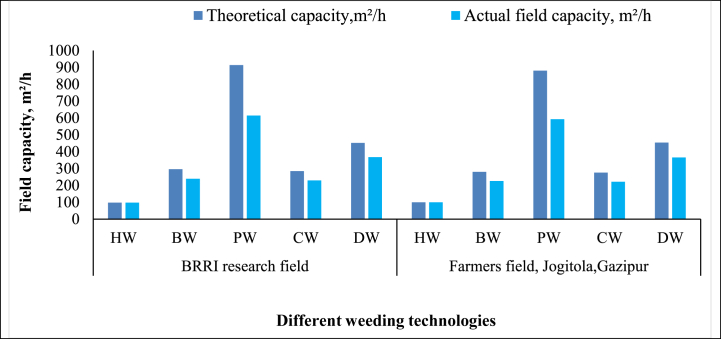
Field capacity of different weeding technologies during first weeding. *Note:* HW = Hand weeding; BW = BRRI weeder; PW = BRRI Power weeder; CW = BRRI Conical weeder and DW= BRRI Double row weeder.

**First weeding:** Various weeding technologies' theoretical and actual field capacities were assessed in the initial weeding operation to determine field efficiency. The actual field capacities for Hand Weeding (HW), the BRRI Weeder (BW), BRRI Power Weeder (PW), BRRI Conical Weeder (CW), and BRRI Double Row Weeder (DW) were recorded at 98, 239, 614, 229, and 368 m^2^/h, respectively, in the BRRI research field. Comparable measurements were taken in a farmer's field in Gazipur, yielding results of 100, 226, 593, 221, and 366 m^2^/h. The BRRI Power Weeder demonstrated a higher actual capacity in both fields, as shown in Fig. 9, which can be attributed to its power-operated mechanism. Conversely, the remaining technologies are manually operated.

**Fig. 10:**
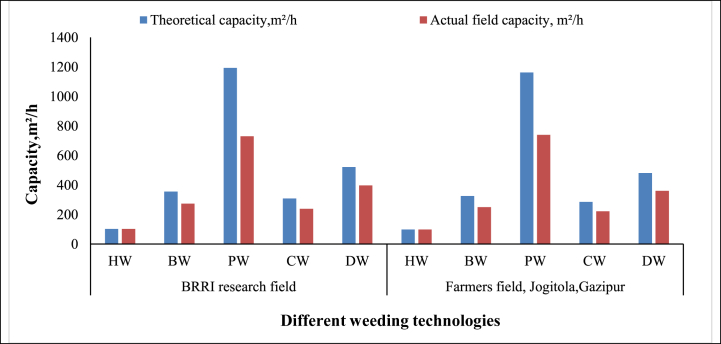
Field capacity of different weeding technologies during second weeding. *Note:* HW = Hand weeding; BW = BRRI weeder; PW = BRRI Power weeder; CW = BRRI Conical weeder and DW= BRRI Double row weeder.

**Second weeding:** Various weeding technologies' theoretical and actual field capacities were measured during the second weeding operation to calculate field efficiency. In the BRRI research field, the actual field capacities for Hand Weeding (HW), BRRI Weeder (BW), BRRI Power Weeder (PW), BRRI Conical Weeder (CW), and BRRI Double Row Weeder (DW) were recorded as 102 m^2^/h, 273 m^2^/h, 730 m^2^/h, 238 m^2^/h, and 397 m^2^/h, respectively (Table 5). In the farmers' fields in Jogitola, Gazipur, the actual field capacities were 98 m^2^/h for HW, 250 m^2^/h for BW, 740 m^2^/h for PW, 222 m^2^/h for CW, and 361 m^2^/h for DW. The Power Weeder demonstrated the highest actual field capacity in both field settings (Fig. 10), attributed to its power-operated mechanism and ability to cover three rows simultaneously, unlike the other manually operated technologies.

### 3.6 Field efficiency

**Fig. 11:**
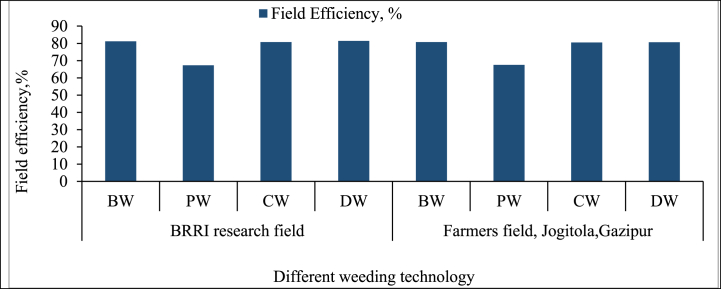
Field efficiency of different weeding technologies. *Note:* HW = Hand weeding; BW = BRRI weeder; PW = BRRI Power weeder; CW = BRRI Conical weeder and DW= BRRI Double row weeder.

**First weeding:** During the first weeding, the “BRRI Weeder (BW)” and “BRRI Conical Weeder (CW)” exhibited the highest field efficiencies, around 80 %, in both the BRRI research field and the farmer's field in Jogitola, Gazipur (Fig. 11). These manually operated weeders are designed to minimize turning time losses, allowing for more continuous and efficient operation. Their compact design and ease of handling make them highly suitable for small to medium-sized fields, where maneuverability and precision are essential. According to Peruzzi et al. [44], such manually operated tools provide an ideal solution for smallholder farmers seeking a balance between efficiency and control. The “BRRI Double Row Weeder (DW)” also performed well, with efficiency slightly lower than BW and CW. Its ability to cover two rows simultaneously provides an advantage regarding the area covered, though its more significant size results in somewhat more time spent on turning and repositioning. As Jasinskas et al. [45] noted, the DW offers a practical option for medium-sized fields where maximizing area coverage is essential without sacrificing control. In contrast, the “BRRI Power Weeder (PW)” showed lower efficiency, around 70 %, despite its higher field capacity. The time lost during turning, especially in smaller or irregularly shaped fields, significantly reduced its overall efficiency. This issue has also been observed in other studies, such as Ousmane et al. [46], where mechanized weeders, while covering larger areas, struggle with frequent repositioning and turning delays. This suggests a need for design improvements to reduce turning time and improve efficiency, particularly in fields with many turns or obstacles.

**Fig. 12:**
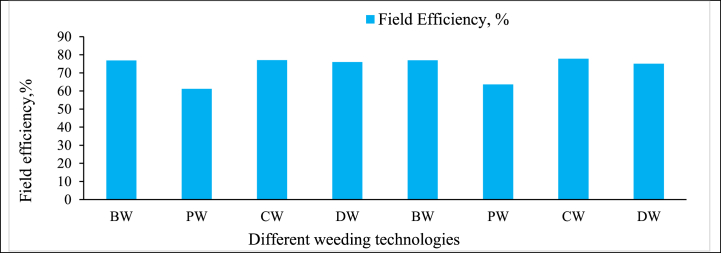
Field efficiency of different weeding technologies during second weeding. Note: HW = Hand weeding; BW = BRRI weeder; PW = BRRI Power weeder; CW = BRRI Conical weeder and DW= BRRI Double row weeder.

**Second weeding:** In the second weeding operation, the “BRRI Weeder (BW)” and “BRRI Conical Weeder (CW)” maintained high field efficiencies of around 77 %, like the first weeding (Fig. 12). Their manual operation allowed for precise control, reducing turning time losses and ensuring efficient weed removal, even as the crops were taller and more delicate. As Bhuiyan et al. [41] point out, these weeders are particularly well-suited for fields that require frequent turns and careful handling to avoid crop damage during the second growth stage. The “BRRI Double Row Weeder (DW)” also performed consistently, with a field efficiency close to 75 %. Its ability to cover two rows simultaneously continued to offer a balance between area coverage and control. However, as in the first weeding, its larger size and complexity in operation resulted in slightly more time lost during turning. Due to significant turning time losses, the “BRRI Power Weeder (PW)” again demonstrated the lowest efficiency, around 61–63 %. Despite its high actual field capacity, the need for careful handling during the second weeding when crops are taller and more prone to damage reduced its overall efficiency. This issue is consistent with findings by Fennimore et al. [34], who reported similar operational delays with mechanized weeders in fields with frequent turns or obstacles. While the PW can cover more ground quickly, its lower field efficiency in the second weeding highlights the challenges of using mechanized tools in small, irregularly shaped fields.

### 3.7 Walking speed

**First weeding:** The data presented indicates a comparative analysis of various weeding technologies' walking speeds in controlled research conditions and actual field settings. The BRRI Power Weeder outperforms other technologies in terms of walking speed, which suggests higher efficiency, particularly in the BRRI research field. However, the Double-row Weeder shows a significant decrease in speed, which could be due to its design catering to precision rather than speed. Compared to the research field, the slight reduction in walking speed across all technologies in the farmer's field could be attributed to actual variables such as terrain irregularities or crop density.

**Walking speed (second weeding):** The walking speed during field operations for different weeding technologies was measured by considering the total operating time, excluding the time lost during turning. The walking speeds for the Blade Weeder (BW), BRRI Power Weeder (PW), BRRI Conical Weeder (CW), and BRRI Double Row Weeder (DW) were recorded as 1777 m/h, 1988 m/h, 1547 m/h, and 1305 m/h, respectively, in the BRRI research field. The walking speeds in the farmers' fields were slightly lower, with BW, PW, CW, and DW achieving 1626 m/h, 1808 m/h, 1427 m/h, and 1204 m/h, respectively (Table 5). These measurements reflect variations in walking speed depending on the weeding technology used and the specific field conditions, influencing overall operational efficiency.

### 3.8 Weeds biomass

The data presented from the paddy fields in Gazipur provides valuable insights into the efficacy of different weeding methods. The variation in weed biomass across the various weeding techniques highlights the importance of selecting the appropriate method for optimal weed management. Hand Weeding, although labor-intensive, shows a relatively low weed biomass, indicating its effectiveness. The BRRI Weeder and BRRI Power Weeder, representing mechanical weeding solutions, demonstrate higher weed biomass, suggesting a trade-off between labor efficiency and thoroughness of weed removal. With their unique designs, the BRRI Conical Weeder and BRRI Double Row Weeder offer alternative approaches, as reflected in their respective weed biomass measurements. These findings underscore the need for careful consideration of weed characteristics, such as type and maturity, when choosing a weeding strategy to ensure the health and productivity of the paddy fields.

### 3.9 Average field capacity

Field capacity across different weeding technologies varied significantly due to factors such as the effective width of the weeder, field dimensions, weed density, operator skill, and soil conditions, including the presence of standing water. The “BRRI Power Weeder (PW)” demonstrated the highest average field capacity of 669 m^2^/h, attributed to its ability to cover multiple rows and its mechanical operation, which enhances weeding speed. However, its fuel consumption (379 ml/h) and higher operating costs present economic and environmental challenges. This is consistent with Sims and Kienzle [47], who found that while mechanized weeders improve labor efficiency, their operational costs make them more suitable for larger-scale farms. In contrast, “Hand Weeding (HW),” with the lowest field capacity (100 m^2^/h), is labor-intensive but offers precision and minimizes crop damage, making it more suitable for small-scale fields or densely planted areas. According to Islam et al. [37], manual weeding remains a viable option for smaller farmers prioritizing control over operational speed. Manually operated weeders like the “BRRI Weeder (BW),” “BRRI Conical Weeder (CW),” and “BRRI Double Row Weeder (DW)” displayed moderate field capacities of 247 m^2^/h, 228 m^2^/h, and 373 m^2^/h, respectively (Table 6). These technologies balance efficiency with cost control, making them suitable for medium-sized fields. Overall, the choice of weeding technology should be driven by field-specific conditions, including field size, weed density, and resource availability. Though efficient in larger fields, mechanized weeders require careful management of fuel consumption and tiller damage, while manual weeders, despite lower coverage capacity, offer better control and are cost-effective for small-scale operations. This aligns with findings from Nath et al. [48], who recommend manual weeders for small farms where labor availability is less constrained.

**Table 6:** Average field capacity and fuel consumption of the weeding technology.

Locations	Time of weeding	Capacity (m^2^/h)					Fuel consumption (ml/h)				
		HW	BW	PW	CW	DW	PW	HW	BW	CW	DW
BRRI research field	1st weeding	98^a^	239^b^	614^c^	229^b^	368^b^	375	–	–	–	–
	2nd weeding	102^a^	273^b^	730^c^	238^b^	397^b^	385	–	–	–	–
Farmer's Field, Jogitola, Gazipur	1st weeding	100^a^	226^b^	593c	221^b^	366^c^	360	–	–	–	–
	2nd weeding	98^a^	250^b^	740^c^	222^b^	361^c^	394	–	–	–	–

Note.

The average value of three replications (N = 3) is presented in the Table.

Superscript letters of alphabets indicate that means followed by the same letter do not significantly differ at p = 0.05.

HW = Hand weeding; BW = BRRI weeder; PW = BRRI Power weeder; CW = BRRI Conical weeder and DW= BRRI Double row weeder.

### 3.10 Cost comparison

Weeding technologies' cost and performance analysis reveals that the “BRRI Power Weeder,” with its high field capacity (669 m^2^/h), is efficient but costly, with a purchase price of Tk 30,000 and high fuel expenses. This aligns with the findings of Ali et al. [49], who noted that mechanized weeders reduce labor costs but are financially viable only for large-scale farms due to high operating and maintenance costs. On the other hand, “manual weeders” like the “BRRI Weeder” and “Conical Weeder” have lower purchase prices (Tk 1,000 and Tk 1,500, respectively) and no fuel costs, making them ideal for smallholders (Table 7). These tools, with moderate field capacities (247 m^2^/h and 228 m^2^/h), offer affordable alternatives for resource-constrained farmers. Studies by Rahaman et al. [50] highlight that manually operated weeders are increasingly preferred in Bangladesh due to their affordability, ease of use, and suitability for small plots. The “Double-row Weeder,” moderately priced at Tk 2,500 with a 373 m^2^/h field capacity, balances cost and efficiency. Anantachar et al. [51] similarly found that this weeder benefits small to medium-sized farms, offering improved labor productivity without the financial burden of mechanized options. Regarding repair and maintenance, the BRRI Weeder, BRRI Conical Weeder, and BRRI Double Row Weeder require no expenses due to their simple technology. In contrast, the BRRI Power Weeder incurs repair and maintenance costs as it is engine-operated. These findings emphasize that while mechanized weeders benefit from more extensive operations, manual weeders are more appropriate for smallholder farmers in Bangladesh due to their lower costs and suitability for small-scale rice fields.

**Table 7:** Cost items and operating costs of different weeders.

Items	Parameter	Amount (DW)		Amount (CW)		Amount (BW)		Amount (PW)	
		Tk	US$	Tk	US$	Tk	US$	Tk	US$
Fixed cost items	Purchase price of weeder (P),	2500	20.83	1500	12.50	1000	8.33	30,000	250
	Salvage value(S), (10 % of P)	250	2.08	150	1.25	100	0.833	300	2.5
	Working life (L), yr	5		5		5		5	
	Average working hours per year	480		480		480		480	
Variable cost items	Labour (Tk or US$/h)	75	0.63	75	0.63	75	0.63	75	0.63
	Repair and maintenance (Tk or US$/yr)	0		0		0		500	4.17
	Field capacity (m^2^/h)	373		228		247		669	
**Calculations**									
Fixed costs	Annual depreciation, D=(P-S)/L Tk or US$/yr	450	3.75	270	2.25	180	1.5	5400	45
	Interest on investment, I=(P + S)/2∗I, where the rate of interest is 12 %	165	1.37	99	0.825	66	0.55	1818	15.15
Total fixed cost	(Tk or US$/yr)	615	5.125	369	3.075	246	2.05	7218	60.15
	(Tk or US$/h)	1.28	0.0106	0.77	6.42 × 10^−3^	0.51	4.25 × 10^−3^	15.04	0.13
	(Tk or US$/ha)	34.31	0.29	33.77	0.28	20.65	0.172	224.81	1.87
Variable cost	Labour (Tk/h)	75	0.63	75	0.63	75	0.63	75	0.63
	Repair and maintenance (Tk/h)	0		0		0		1.37	0.0114
Total variable cost	(Tk or US$/h)	75	0.63	75	0.63	75	0.63	76.37	0.64
	(Tk or US$/ha)	2,010.72	16.76	3,289.47	27.41	3,036.44	25.30	1141.55	9.51
Operating cost	(Tk or US$/h)	76.28	0.64	75.77	0.631	75.51	0.63	91.41	0.76
	(Tk or US$/ha)	2,045.04	17.04	3,323.24	27.69	3,057.09	25.48	1366.37	11.39

**Note:** Average workday = 8 h at 0.037 ha/h; Labor/operator charge = 600 Tk/day, 1 US$ = 120 BD TK.

BW = BRRI weeder; PW = BRRI Power weeder; CW = BRRI Conical weeder and DW= BRRI Double row weeder.

## 4 Sustainable weed management for rice in Bangladesh: aligning with SDGs 2 & 12

The findings of this study align with the United Nations' Sustainable Development Goals (SDGs), particularly Goal 2: Zero Hunger and Goal 12: Responsible Consumption and Production. Rice, as the staple food crop in Bangladesh, plays a critical role in ensuring food security and economic stability, occupying 75 % of the country's agricultural land. Efficient weed management is essential, with several advantages within cropping systems to maximize rice yields and minimize losses ranging from 15 % to 60 % due to competition from weeds. The study's evaluation of five weeding technologies underscores the potential for sustainable farming practices that reduce labor dependency and enhance productivity. Mechanical weeders like the BRRI Power Weeder (PW) demonstrate significant labor savings, while manually operated weeders offer safer, more precise options for smaller-scale fields. By promoting technologies that optimize operational efficiency and safeguard crops, this research supports the sustainable intensification of rice production, addressing food security while minimizing environmental impacts. Integrating such innovations with broader agricultural strategies will be key to achieving long-term sustainability and resilience in Bangladesh's rice sector, contributing to the SDGs' overarching mission of eradicating hunger and fostering responsible agricultural practices.

## 5 Conclusion and way forward

This study evaluated the operational effectiveness of various mechanical weeders in rice fields in Bangladesh, focusing on field capacity, tiller damage, field efficiency, and fuel consumption. The BRRI Power Weeder (PW) had the highest field capacity (669 m^2^/h), making it efficient regarding the area covered. However, it also caused more significant tiller damage and reduced field efficiency due to time lost in turning. On the other hand, manually operated weeders like the BRRI Weeder (BW), BRRI Conical Weeder (CW), and BRRI Double Row Weeder (DW) offered moderate field capacities and better crop safety, with higher field efficiency attributed to lower turning losses. Although labor-intensive, Hand Weeding (HW) provided the best precision and minimal crop damage and was suited for smaller fields or densely planted areas. The findings suggest that power-operated weeders can increase efficiency but require careful management to reduce crop damage.

Successful weed management in diverse agricultural settings requires a multi-pronged approach. Farmers must carefully select weeding technologies based on their needs, considering field size, crop density, and available resources. While power-operated weeders excel in large fields, offering significant time and labor savings, their effectiveness hinges on minimizing crop damage through refined designs and careful operation. Manually operated weeders are generally more suitable for smaller fields or dense plantings, prioritizing precision and crop safety. Concurrent research efforts are essential. Extension workers must take proactive measures to disseminate and promote the adoption of weeding technologies at the field level to achieve the SDG and enhance rice production with minimal health risks. Researchers should prioritize refining power-operated weeder designs to minimize tiller damage and enhance field efficiency, considering factors like soil conditions, weed types, and crop layouts. Furthermore, policymakers must incentivize the adoption of sustainable weeding technologies through subsidies, training programs, and support for research and innovation. This collaborative approach will foster the development and dissemination of cost-effective and user-friendly mechanical weeders, ensuring long-term sustainability and enhancing agricultural productivity for smallholder farmers.

## CRediT authorship contribution statement

**Subrata Paul:** Writing – review & editing, Writing – original draft, Validation, Supervision, Resources, Project administration, Methodology, Investigation, Formal analysis, Data curation, Conceptualization. **Bidhan Chandra Nath:** Writing – review & editing, Methodology, Formal analysis. **Md. Durrul Huda:** Supervision, Investigation, Funding acquisition. **Md. Golam Kibria Bhuiyan:** Supervision, Methodology. **Haimonti Paul:** Validation, Investigation, Data curation.

## Ethics statement

The research included photographs of individuals, with informed consent obtained from all participants before image capture and use. The study adhered to ethical guidelines, ensuring participants were fully informed about the research purpose, image use, and their right to withdraw consent. The Farm Machinery and Postharvest Technology Division, Bangladesh Rice Research Institute Ethics Committee reviewed and approved the study protocol.

## Data availability statement

The data presented in this study is available on request from the corresponding author.

## Funding

This research received no external funding.

## Declaration of competing interest

The authors declare that they have no known competing financial interests or personal relationships that could have appeared to influence the work reported in this paper.
